# Bridging trauma and eating disorders: the role of loneliness

**DOI:** 10.3389/fpsyt.2024.1500740

**Published:** 2024-12-10

**Authors:** Paolo Meneguzzo, Anna Marzotto, Barbara Mezzani, Fabio Conti, Luca Maggi, Patrizia Todisco

**Affiliations:** ^1^ Department of Neuroscience, University of Padova, Padua, Italy; ^2^ Padova Neuroscience Center, University of Padova, Padua, Italy; ^3^ Eating Disorder Unit, Casa di Cura “Villa Margherita” – KOS Group, Vicenza, Italy; ^4^ Eating Disorders Unit, Casa di Cura Villa dei Pini – KOS Group, Florence, Italy; ^5^ Eating Disorders Unit, Casa di Cura Villa Armonia – KOS Group, Rome, Italy; ^6^ Eating Disorders Unit, Casa di Cura Ville di Nozzano – KOS Group, Lucca, Italy

**Keywords:** trauma, loneliness, mediation, child trauma, psychopathology, eating disorders

## Abstract

**Introduction:**

Eating disorders (EDs) are complex and often linked to traumatic childhood experiences. While childhood trauma is known to increase the risk of EDs, the role of loneliness remains underexplored. This study investigates whether loneliness mediates the relationship between childhood trauma and ED symptoms.

**Methods:**

A total of 230 individuals with EDs completed the Childhood Trauma Questionnaire, the UCLA Loneliness Scale, and the Eating Disorders Examination Questionnaire. Mediation analysis was conducted to assess if loneliness mediates the relationship between childhood trauma and ED severity.

**Results:**

Childhood trauma significantly predicted higher levels of loneliness (p < 0.001), which was associated with more severe ED symptoms (p = 0.001), with age and BMI as covariates. Mediation analysis showed loneliness partially mediated the relationship between childhood trauma and ED severity (indirect effect b = 0.003, 95%CI [0.001, 0.006]).

**Conclusion:**

Loneliness partially mediates childhood trauma and ED symptoms, highlighting the need to address loneliness in treatment to mitigate the impact of childhood trauma on ED severity. These findings suggest the possible role of social connection-focused interventions in ED care and contribute to understanding the mechanisms underlying the development of EDs. Future research should explore additional mediators and moderators to provide a more comprehensive perspective.

## Introduction

Eating disorders (EDs), which encompass conditions such as anorexia nervosa (AN), bulimia nervosa (BN), and binge-eating disorder (BED), represent a significant public health concern due to their profound physical, psychological, and social consequences (1–3). These disorders are marked by persistent disturbances in eating behavior, accompanied by distressing thoughts and emotions related to food, body weight, and shape (1). In line with the biopsychosocial model, this complex etiology underscores the importance of considering social and environmental factors, alongside biological and psychological influences, in understanding and addressing EDs (4). Among environmental factors, childhood traumas have emerged as a critical area of focus, given their long-lasting impact on mental health (5, 6). Recent studies report varying prevalence rates of EDs, with lifetime prevalence ranging from 2.2% for men to 8.4% for women (7, 8). These rates vary by region, with higher point prevalence observed in America (4.6%) and Asia (3.5%) compared to Europe (2.2%), suggesting that eating disorders are highly prevalent worldwide, particularly among women, and that the incidence of these disorders has been increasing over time. The distribution of ED varies widely, with AN typically being more prevalent in clinical settings, particularly in inpatient facilities, while BN and BED are often more common in outpatient or community samples (9). The prevalence of each diagnosis is also influenced by factors such as gender, age, and geographical region (10).

Traumatic childhood experiences, including physical, emotional, and sexual abuse, as well as neglect and household dysfunction, have been extensively documented as significant risk factors for the development of various psychological disorders (11, 12). These early adversities disrupt the normal developmental trajectory, leading to maladaptive coping mechanisms and alterations in neurobiological stress response systems (13). Research has consistently shown that individuals with a history of childhood trauma are at a heightened risk for developing eating disorders, as these individuals may turn to disordered eating behaviors as a means to exert control, manage negative emotions, or dissociate from traumatic memories (14). Recently, various studies have suggested the possible presence of an echophenotype in individuals with eating disorders who have a history of trauma, indicating more severe clinical conditions and physical changes that constitute a different and specific manifestation of the disorder (15, 16).

Social support, a key element in the biopsychosocial model, plays a crucial role in mental health by providing emotional resources that help individuals manage stress and adversity (17). Supportive relationships can buffer the impact of trauma, fostering resilience and promoting healthier coping strategies (18, 19). Conversely, a lack of social support or experiences of social isolation can heighten vulnerability to psychological distress, intensifying the risk of developing or worsening symptoms of mental health conditions, including eating disorders (18, 20).

Loneliness and social isolation also play critical roles in eating disorders across diagnostic categories. Social isolation, defined as a lack of meaningful social interactions, is a frequent experience among individuals with eating disorders and can heighten the sense of disconnection from others (21). This is particularly relevant given that social withdrawal is common in ED populations, where shame and stigma surrounding eating behaviors may lead to avoidance of social situations (22, 23). Such isolation exacerbates feelings of loneliness—a perceived deficit in social connections—and has been associated with the maintenance and severity of eating disorder symptoms across various diagnoses, including AN, BN, and BED (24–26). In addition to the effects of childhood trauma, the role of loneliness as a mediation feature has gained increasing attention in the context of mental health outcomes, in different disorders and different contexts (27–29). Individuals who have experienced childhood trauma often report higher levels of loneliness, stemming from difficulties in forming and maintaining secure attachments and trusting relationships (30). This chronic sense of isolation and social disconnection can exacerbate the psychological distress associated with eating disorders, creating a vicious cycle that reinforces maladaptive eating behaviors (31). Furthermore, loneliness has recently been identified as a potential factor that may contribute to the worsening of physical conditions in people with eating disorders, calling for further investigations (24).

The intersection of childhood trauma, loneliness, and eating disorder psychopathology represents a compelling area for investigation. While the link between childhood trauma and EDs has been well-established (32), the mediating role of loneliness in this relationship remains underexplored. Understanding how loneliness mediates this relationship can provide crucial insights into the underlying mechanisms that contribute to the development and persistence of eating disorders. This knowledge can inform the design of targeted therapeutic interventions aimed at alleviating loneliness, thus potentially mitigating the impact of childhood trauma on the symptoms of eating disorders.

This study aims to explore the mediating effect of loneliness in the relationship between traumatic childhood experiences and eating disorder psychopathology. By employing a mediation analysis approach, we seek to elucidate the pathways through which early adverse experiences influence EDs symptoms, highlighting the critical role of loneliness. Our findings are expected to contribute to the growing body of literature regarding the etiology of eating disorders and offer practical implications for enhancing treatment strategies to address loneliness in individuals with a history of childhood trauma.

## Methods

### Participants

The study involved 230 individuals diagnosed with eating disorders, recruited from four national clinics, specialized in the treatment of these disorders, at the onset of their inpatient treatment. Diagnoses were established by experienced eating disorder clinicians using the semi-structured clinical interview based on DSM-5 criteria, which is standard in clinical practice. The high proportion of AN participants is due to the recruitment setting within inpatient clinics. The exclusion criteria were the presence of a severe psychiatric acute comorbidity like psychosis or mania, or the presence of cognitive deficits. All participants identified as cisgender, with the majority being white (95.9%). All participants filled out a written informed consent to participate at the study.

### Measures

Data were collected within one week of admission for the EDs group as part of routine service evaluation. The survey package included the Eating Disorders Examination Questionnaire (EDE-Q), the revised University of California Los Angeles Loneliness Scale (UCLA), and the Childhood Trauma Questionnaire (CTQ). All three instruments demonstrated good internal consistency (α > 0.80).

The EDE-Q is a 28-item self-report measure designed to assess the severity of eating disorder psychopathology (33). Participants respond using a 7-point Likert scale, with higher scores indicating greater severity of symptoms. The EDE-Q provides a global score and four subscales: restraint, eating concerns, shape concerns, and weight concerns.

The UCLA Loneliness Scale is a 20-item questionnaire that evaluates subjective feelings of loneliness and social isolation (34). Responses are rated on a 4-point Likert scale, with higher scores reflecting elevated levels of loneliness. This scale is widely used and validated for assessing loneliness in various populations.

The CTQ is a retrospective self-report inventory that measures the extent of traumatic experiences during childhood (35). It includes 28 items, each rated on a 5-point Likert scale, encompassing a total score and five subscales: emotional abuse, physical abuse, sexual abuse, emotional neglect, and physical neglect. Higher scores indicate greater levels of reported childhood trauma.

### Statistical plan

The statistical analysis involved a comprehensive examination of the sample, including descriptive statistics such as means and standard deviations for continuous variables, and rates for the prevalence of traumatic experiences based on the CTQ. Mediation analysis was conducted using the SPSS PROCESS macro-extension (version 3.5), specifically Model 4 (36). In this analysis, the CTQ total score was utilized as the independent variable, with the UCLA Loneliness Scale score as the mediator and the EDE-Q total score as the dependent variable. To assess the mediation effect, bootstrapping with 5,000 samples was performed to estimate the indirect effects, with bias-corrected confidence intervals set at 95%. Bootstrapping involves repeatedly sampling from the data to calculate indirect effects and their confidence intervals, providing a more accurate and reliable estimate by simulating thousands of potential scenarios (37). The mediation model was evaluated including age and BMI as covariates. Additionally, the Sobel test was employed as a confirmatory analysis to validate the overall indirect effect. The Sobel test is a traditional method to check whether a mediator significantly explains the relationship between the independent and dependent variables, offering a straightforward calculation of statistical significance (38). Statistical significance was determined at an alpha level of p < 0.05 for all analyses, executed using IBM SPSS Statistics 25.0 (SPSS, Chicago, IL, United States).

## Results

The sample consisted of 206 women (89.4%) and 24 men (10.6%). The mean age was 26.58 ± 11.82 years, with a mean BMI of 22.53 ± 12.02 kg/m^2^, and an average duration of the disorder of 6.90 ± 8.02 years. Diagnoses within the sample included 99 individuals with restrictive anorexia nervosa (42.8%), 34 with binge-purge anorexia nervosa (14.7%), 36 with bulimia nervosa (15.3%), 47 with binge eating disorder (20.0%), and 14 with other specified feeding and eating disorders (6.2%). Most participants lived with their parents (n = 165, 71.7%), while fewer lived alone (n = 35, 15.2%) or with a partner (n = 30, 13.0%). Regarding employment status, the majority reported having a stable occupation (n = 84, 36.5%), a significant proportion were unemployed (n = 84, 36.5%), and 56 were students (24.3%). EDEQ global score was 4.12 ± 1.31, CTQ global score was 44.12 ± 17.97, and UCLA Loneliness score was 49.00 ± 12.16. See **Table 1** for details.

**Table 1:** Demographic and clinical characteristics of the sample.

	ED N=	ANr n = 99	ANbp n = 34	BN n = 36	BED n = 47	OSFED n = 14
Gender						
Female Male	206 24	97 2	34 0	36 0	31 16	8 6
Age, years	26.58 11.82	24.56 10.18	24.71 8.59	23.78 7.57	35.23 16.15	23.64 8.54
BMI, kg/m^2^	22.53 10.02	15.05 1.62	16.19 1.31	21.98 3.88	43.81 9.28	20.93 1.89
Duration of the disorder, years	6.90 8.02	5.50 7.27	6.54 7.64	8.30 7.44	8.68 9.82	8.90 8.24
Living condition						
With parents With partner Alone	165 30 35	82 9 8	25 3 6	25 4 7	23 10 14	10 4 0
Employment status						
Occupied Unemployed Students	84 84 56	28 38 33	13 12 9	8 21 7	34 9 4	7 4 3
EDEQ global score	4.12 1.31	4.02 1.35	4.34 1.35	4.77 1.00	3.48 1.18	4.82 0.93
CTQ global score	44.12 17.97	36.20 11.73	44.82 21.07	50.86 13.29	50.34 20.38	60.14 23.66
UCLA loneliness scale	49.00 12.16	48.79 12.00	49.82 10.97	48.67 14.08	48.79 12.71	50.00 10.21

The table reports the mean and standard deviation for continuous variables and raw counts for categorical variables. ED, eating disorder; AN, anorexia nervosa; r, restrictive; bp, binge-purge; BN, bulimia nervosa; BED, binge eating disorder; OSFED, other specified feeding and eating disorder; BMI, body mass index; EDEQ, eating disorder examination questionnaire; CTQ, childhood trauma questionnaire.

### Mediation analyses

The analysis indicated that the CTQ total score significantly predicted UCLA, b = 0.141, p < 0.001, suggesting that higher levels of childhood trauma are associated with higher UCLA Loneliness scores. UCLA, in turn, significantly predicted EDEQ global score, b = 0.021, p = 0.001, indicating that greater UCLA Loneliness scores are related to more severe eating disorder symptoms.

**Figure 1:**
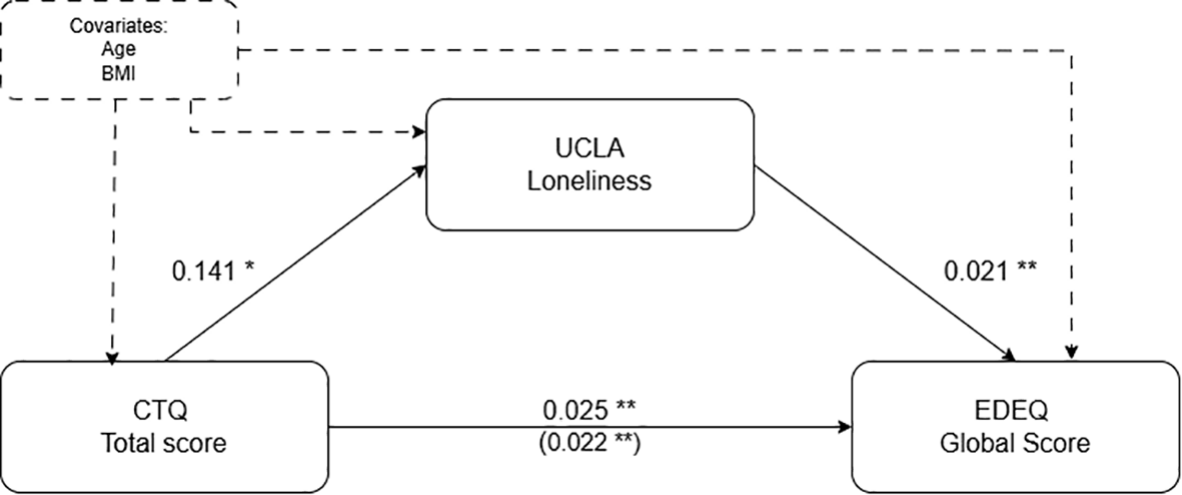
Standardized regression coefficients for the relationship between childhood trauma (CTQ total score) and eating disorder psychopathology (EDEQ global score) as mediated by UCLA Loneliness, corrected for age and BMI. The regression coefficient of the mediation is in parentheses. *p < 0.01; **p <= 0.001.

The total effect of CTQ total score on EDEQ global score was significant, b = 0.025, p < 0.001. The direct effect of CTQ total score on EDEQ global score, after accounting for UCLA, was also significant, b = 0.022, p < 0.001. The indirect effect of CTQ total score on EDEQ global score through UCLA Loneliness was b = 0.003, with a 95% bootstrap confidence interval of [0.001, 0.006], which does not include zero, indicating a significant mediation effect. The completely standardized indirect effect was b = 0.040 (95% CI [0.009, 0.082]), further confirming that UCLA Loneliness significantly mediates the relationship between total childhood trauma and eating disorder symptoms. These results suggest that the effect of childhood trauma on eating disorders is partially mediated by UCLA. See **Figure 1** for the mediation analysis graphical representation and **Table 2** for details.

**Table 2:** Regression analysis of eating disorders on childhood trauma and loneliness.

Predictors	B	SE	p	95% CI	R^2^
Model 1					
CTQ total score	0.025	0.044	< 0.001	0.016, 0.034	0.235
Age	-0.038	0.007	< 0.001	-0.051, -0.024	
BMI	-0.016	0.007	0.019	-0.029, -0.003	
Model 2					
CTQ total score	0.022	0.004	< 0.001	0.013, 0.031	0.275
UCLA loneliness	0.021	0.006	0.001	0.008, 0.033	
Age	-0.036	0.007	< 0.001	-0.049, -0.023	
BMI	-0.015	0.007	0.031	-0.028, -0.001	
	Effect	SE	p	95% CI	
Total effect	0.025	0.004	< 0.001	0.016, 0.034	
Direct Effect	0.022	0.004	< 0.001	0.013, 0.031	
Indirect Effect	0.003	0.001		0.001, 0.006	

Model 1 includes CTQ total score as the predictor variable, with EDE-Q total score as the dependent variable. Model 2 includes CTQ total score and UCLA Loneliness as predictor variables. This model tests the effect of childhood trauma on eating disorder symptoms, while also accounting for the mediating role of loneliness (UCLA), which significantly predicts eating disorder severity. The increase in R² from Model 1 to Model 2 indicates a stronger model fit when UCLA loneliness is included. The table reports Models corrected for age and BMI.

EDEQ, eating disorder examination questionnaire; CTQ, childhood trauma questionnaire.

Sobel test corroborated the results of the mediation analysis showing a significant mediation with Sobel test statistic = 2.355 and p = 0.018.

## Discussion

The present study explored the mediating effect of loneliness on the relationship between traumatic childhood experiences and eating disorder psychopathology. The findings reveal that loneliness significantly mediates the relationship between childhood trauma and the severity of eating disorder symptoms. This has profound clinical implications, as it provides insights into the underlying mechanisms that contribute to the development and persistence of eating disorders and suggests avenues for targeted therapeutic interventions. Previous research has established a link between childhood trauma and eating disorders (4, 12), but the mediating role of loneliness has been underexplored. Our findings extend this understanding by demonstrating that loneliness significantly mediates this relationship.

This study addresses a critical gap in the literature by examining the mediating role of loneliness, providing a more nuanced understanding of how early adverse experiences can lead to disordered eating behaviors through the intermediary of social isolation. Indeed, loneliness is pointed out as a crucial element in the pathway from childhood trauma to eating disorder symptoms. This finding aligns with the theoretical framework that traumatic childhood experiences disrupt normal developmental trajectories, leading to maladaptive coping mechanisms and alterations in neurobiological stress response systems (39, 40). From a clinical perspective, this underscores the importance of addressing loneliness in therapeutic settings to disrupt this pathway. Otherwise, interventions might be not useful for the improvement of patients’ health.

Moreover, the mediating role of loneliness suggests that interventions aimed at reducing feelings of isolation and enhancing social connections could be effective in treating individuals with eating disorders who have a history of childhood trauma. In this perspective, Cognitive Behavioral Therapy (CBT) and Interpersonal Therapy (IPT), which focus on improving social skills and building meaningful relationships, may be particularly beneficial (41–44). Also, specific remediation therapy interventions like Cognitive Remediation and Emotional Skills Training (CREST) might be considered an effective add-on treatment with these specific goals due to their efficacy in implementing specific interpersonal features (45).

The results corroborate recent studies that have identified loneliness as a significant factor in the worsening of physical and psychological conditions, including eating disorders (24, 31, 46). By empirically demonstrating the mediating role of loneliness, this study reinforces the importance of social connections in mental health and eating disorder psychopathology specifically (47, 48). We found evidence of partial mediation, supporting the idea that childhood maltreatment and loneliness have both combined and independent effects. However, the partial nature of these relationships suggests opportunities for future research to explore additional mediators and moderators. Factors such as resilience, social support, and coping strategies could be examined to provide a more comprehensive understanding of the complex interplay between childhood trauma, loneliness, and eating disorders. Longitudinal studies could also investigate the temporal dynamics of these relationships, offering further insights into causality and optimal intervention timing.

Finally, the findings emphasize the need for personalized treatment plans that take into account the patient’s history of childhood trauma and current levels of loneliness (49). Clinicians should consider incorporating assessments for loneliness and trauma history into their diagnostic process to identify patients who may benefit from targeted interventions aimed at improving social connections. This tailored approach can lead to more effective treatment outcomes. Indeed, there is growing evidence that both loneliness and traumatic events have specific disruptive effects on life trajectories, increasing both psychological and physical symptoms (50). From a preventive perspective, early interventions targeting loneliness in children who have experienced trauma have been reported to be effective in reducing the long-lasting negative effects on physical and mental health (51–53). This approach could also reduce the risk of developing eating disorders later in life (54, 55). School-based programs and family interventions that promote healthy attachment and social support could play a crucial role in preventing the onset of eating disorders among at-risk populations (55–57).

This study prioritized mediation analysis to explore the mechanisms underlying the relationships between childhood trauma, loneliness, and ED symptoms. The decision not to examine moderation was guided by our aim to identify potential indirect pathways rather than the interaction effects or conditions that might influence these relationships (58). However, investigating moderation could offer valuable insights into how individual differences or contextual factors, such as gender, cultural background, or severity of symptoms, might shape these associations (59)-all aspects that ED research needs (60). Future research should consider incorporating moderation analyses to provide a more comprehensive understanding of the complexity of these interactions.

### Limitations

Several limitations of this study should be acknowledged. First, the cross-sectional design precludes any definitive conclusions about the causal relationships between childhood trauma, loneliness, and eating disorder symptoms. Longitudinal studies are needed to establish the temporal sequence of these variables. Second, the reliance on self-report measures may introduce response biases, such as social desirability or recall biases, which could affect the accuracy of the data. Third, the sample predominantly consisted of individuals identifying as cisgender and white, limiting the generalizability of the findings to more diverse populations. Additionally, the mixed sample, which includes individuals with various eating disorder diagnoses, may represent a limitation, as these disorders differ significantly in their phenotypic expression and in their associations with childhood trauma—particularly as bulimic variants are more frequently associated with histories of physical and sexual abuse. Moreover, the higher representation of individuals with anorexia nervosa in our sample, likely due to the enrollment criteria of inpatient facilities, may limit the generalizability of the findings to those with other eating disorder diagnoses, as inpatient settings tend to prioritize patients with more severe forms of AN. Furthermore, the inclusion of male participants could introduce a confounding variable, as gender may affect the presentation and correlates of eating disorder symptoms. Moreover, while loneliness was a central variable in our study, we did not assess depressive symptoms, which may be associated with loneliness and could provide a broader understanding of its role in eating disorders; this is a key limitation and an area for further exploration in future studies. Finally, the study did not account for other potential mediators or moderators that could influence the relationship between childhood trauma and eating disorders, such as genetic factors, personality traits, or other environmental influences.

## Conclusion

This study underscores the significant mediating role of loneliness in the relationship between traumatic childhood experiences and eating disorder psychopathology. These findings highlight the importance of addressing loneliness in therapeutic interventions to mitigate the impact of childhood trauma on eating disorder symptoms. By advancing the understanding of the underlying mechanisms and suggesting targeted therapeutic approaches, this study contributes to the development of more effective and personalized treatment strategies. Additionally, it fills a critical gap in the literature and sets the stage for future research to further elucidate the complex relationships between childhood trauma, loneliness, and eating disorders.

## Data Availability

The raw data supporting the conclusions of this article will be made available by the authors, without undue reservation.
